# The prognostic and immune significance of PLBD1 in pan-cancer and its roles in proliferation and invasion of glioma

**DOI:** 10.7150/jca.96365

**Published:** 2024-05-20

**Authors:** Minghao Wei, Gaoyang Zhou, Lian Chen, Yufu Zhang, Wei Ma, Li Gao, Guodong Gao

**Affiliations:** 1 Department of Neurosurgery, Tangdu Hospital, the Fourth Military Medical University, Xi'an, Shaanxi, 710038, China.; 2 Department of Neurosurgery, the Shengjing Hospital of China Medical University, Shenyang, Liaoning, 110001 China.; 3 Department of Neurosurgery Ward II, the Second Affiliated Hospital of Shaanxi University of Chinese Medicine, Xianyang, Shaanxi, 712046, China.

**Keywords:** PLBD1, Glioma, Immunotherapy, Immune microenvironment, Pan-cancer

## Abstract

Cancer is a destructive disease and is currently the leading cause of major threats to human health. PLBD1 is a transcription factor that regulates phospholipid metabolism, but its role in tumors is unknown. We assessed pan-cancer expression, methylation, and mutation data of PLBD1 by multiple databases to investigate its clinical prognostic value. In addition, we examined the pan-cancer immunological signature of PLBD1, particularly in gliomas. Furthermore, we assessed the impact of PLBD1 knockdown on the proliferation and invasive capacity of glioma cells by *in vitro* experiments. Our results suggest that PLBD1 is highly expressed in multiple types of cancers, and it can serve as an independent prognostic factor for gliomas. In addition, we found that the epigenetic alterations of PLBD1 were highly heterogeneous in a variety of cancers, including gliomas, and that its high methylation was associated with poor prognosis in a broad range of cancers. Immunological profiling demonstrated that PLBD1 was significantly associated with immune cell infiltration and multiple immune checkpoints in gliomas and is a potential biomarker for gliomas. Furthermore, cellular experiments showed that knockdown of PLBD1 significantly inhibited the proliferation and invasive ability of glioma cells. In conclusion, PLBD1 is a potential tumor prognostic biomarker and immunotherapeutic target that plays a crucial role in glioma cell proliferation, invasion and immunotherapy.

## 1. Introduction

The prevalence of malignant tumors has increased at an alarming rate over the past few decades and has become the leading cause of human mortality and a major burden on global public health 1. In recent years, as noted by the American Cancer Society, cancer mortality rates have continued to decline, owing to advances made in the field of cancer treatment 2, 3. Gliomas are the most common tumors in the central nervous system, accounting for more than 80% of cases, with glioblastoma multiforme (GBM) being the most malignant and aggressive 4. Although progress has been made in the comprehensive treatment of gliomas, including surgery, radiotherapy and chemotherapy, the overall prognosis remains poor, and the long-term survival rate is still very low 5.

In recent years, cancer immunotherapy has become a prominent cancer treatment, such as immune checkpoint-targeted monoclonal antibody and chimeric antigen receptor T-cell therapy 6. However, a significant proportion of cancer patients still show resistance to these therapies and fail to achieve a durable response 7. Moreover, single cancer-targeting studies limit our understanding of the multifaceted nature of cancer-associated genes and traits. Therefore, studying and searching for new immunomodulatory genes from a macroscopic "pan-cancer" perspective is crucial for developing more precise immunotherapy regimens.

Phospholipase B Domain-Containing Protein 1 (PLBD1) is a key transcription factor regulating phospholipid metabolism 8. The study suggests that the RNA expression level of PLBD1 in peripheral blood can be a novel and independent predictor of left ventricular insufficiency after acute myocardial infarction, ischemic stroke, and aneurysm subarachnoid hemorrhage 9-11. The results of existing studies suggest that PLBD1 has value as a diagnostic and prognostic marker for pancreatic ductal adenocarcinoma 12. Several studies have suggested that risk models based on multiple genes such as PLBD1 may predict the prognosis of glioma patients and reflect the immunological characteristics of glioma 13, 14. However, the correlation between PLBD1 and tumor including glioma has not been thoroughly evaluated.

In this study, we explored the expression and prognosis of PLBD1 in various cancers using public databases and confirm its high expression levels in glioma clinical samples. Furthermore, the association between PLBD1 and genomic alterations, prognosis, Gene Set Enrichment Analysis (GSEA) and immune cell infiltration analysis suggests that PLBD1 has the potential to be a promising biomarker for immunotherapy. In addition, molecular biology experiments were performed in glioma cell lines to further validate the oncogenic function of PLBD1. In conclusion, PLBD1 may serve as a potential therapeutic target for cancer therapy, indicating immune infiltration and poor prognosis of cancer patients.

## 2. Materials and Methods

### 2.1 Data collection

The mRNA expression profiles and clinical data for 33 cancers were obtained from The Cancer Genome Atlas (TCGA) database (https://portal.gdc.cancer.gov/) and Clinical proteomic tumor analysis Consortium (CPTAC). The mRNA expression profiles for normal tissues were downloaded from the Genotype-Tissue Expression (GTEx) database (https://www.gtexportal.org/ home/) and the Human Protein Atlas (HPA) database (https://www.proteinatlas.org/). Cell line gene expression matrix of tumors was acquired from the Cancer Cell Line Encyclopedia dataset (CCLE, https://portals.broadinstitute.org/ccle/about). The glioma datasets CGGA325 and CGGA693 were derived from the Chinese Glioma Genome Atlas (CGGA) database (http://www.cgga.org.cn/) 15. Finally, tumor mutation load (TMB) and microsatellite instability (MSI) data were obtained from the previous studies 16, 17.

### 2.2 Patient specimens

Paraffin-embedded glioma samples (acquired from 2016/7 to 2017/1) with WHO II (n = 10), WHO III (n = 10), WHO IV (n = 10), and frozen glioma samples (obtained from 2020/1 to 2020/6) were obtained from the Department of Neurosurgery, Tangdu Hospital (Xi'an, China). The normal brain tissues were obtained from the normal cortex in LGG patients. Inclusion criteria for patients with glioma: 1) patients over 18 years of age; 2) pathological diagnosis of glioma and not having received any radiotherapy, chemotherapy, or other treatment prior to surgery; and 3) signing an informed consent form. Exclusion criteria included having acute myocardial infarction, heart failure, liver disease (cirrhosis, fatty liver, and viral hepatitis, etc.), other malignancies, peripheral autoimmune diseases, and hematologic diseases. The detailed information is shown in Supplementary Table 1. All patients had signed a written informed consent, and the protocol was also approved by the Ethics Committee of Tangdu Hospital of the Fourth Military Medical University.

### 2.3 The differential expression, prognosis, and epigenetic analysis of PLBD1 in pan-cancer, glioma and single-cell

We analyzed PLBD1 expression in normal tissues using HPA and GTEx data, analyzed PLBD1 expression levels in cancer cell lines using CCLE data, and analyzed PLBD1 expression in single cells using Tumor Immunity Single Cell Centre (TISCH) data (http://tisch.comp-genomics.org/). Differential expression of PLBD1 in tumor tissues and normal tissues was compared using TCGA, CPTAC or integrated TCGA and GTEx expression profiles. PLBD1 expression and prognosis were analyzed using TCGA, CGGA325 and CGGA693. Samples from 33 cancer types were divided into high and low expression groups based on the median expression of PLBD1. Afterwards, survival time and survival status were compared between the two groups using the R software package "survival." P values of Kaplan-Meier curves and hazard ratios (HR with 95% confidence intervals [CI]) were analyzed by log-rank tests. Univariate cox regression analysis was used to assess the value of PLBD1 as an independent prognostic indicator.

We assessed the characteristics of PLBD1 methylation in pan-cancer tissues with the TCGA methylation module. In addition, we analyzed the effect of methylation and copy number variation (CNV) on the survival states of patients. The GSCA (http://bioinfo.life.hust.edu.cn/web/GSCA/) platform was used to investigate the correlation between PLBD1 mRNA expression and copy number variation (CNV) and the extent of PLBD1 methylation in different tumors.

### 2.4 The relationship between PLBD1 and pan-cancer immune cell infiltration and immunotherapy response

We used R package “ESTIMATE v 1.0.13” to calculate the stromal, immune, and ESTIMATE scores of each tumor sample 18. 7 methods, including single-sample Gene Set Enrichment Analysis (ssGSEA), Cell-type Identification by Estimating Relative Subsets of RNA Transcripts (CIBERSORT), CIBERSORT-ABS, Tumor IMmune Estimation Resource (TIMER), Estimating the Proportion of Immune and Cancer cells (EPIC), XCell and Microenvironment Cell Populations (MCP)-counter, to determine the correlation between PLBD1 and pan-cancer immune cells infiltration. The response of PLBD1 high- and low-expression groups to programmed cell death protein 1 (PD-1) and cytotoxic T-lymphocyte associated protein 4 (CTLA4) immunotherapy were also evaluated from GSE78220, GSE91061 and Imvigor210.

### 2.5 The differential expression, functional enrichment and immunological characteristic between PLBD1 high- and low- expression group in glioma

Based on the previous study 19, we acquired 122 immune modulators and assessed their association with PLBD1 mRNA expression in LGG and GBM. Anti-cancer immune status describes the diverse events of the cancer immune cycle. We utilized the anti-cancer immune status at 7 different stages of the cancer immune cycle by using the Tracking Tumor Immunophenotype (TIP) database (http://biocc.hrbmu.edu.cn/TIP/)^20^, which contained the release of cancer cell antigens (step 1), cancer antigen presentation (step 2), priming and activation (step 3), trafficking of immune cells to tumors (step 4), infiltration of immune cells into tumors (step 5), recognition of cancer cells by T cells (step 6), and killing of cancer cells (step 7). We used CIBERSORT to calculate the level of immune cells infiltration in glioma. We studied the differential expression of PLBD1 high- and low-expression groups in gliomas using the R package "Limma". In addition, we made use of the R package "ClusterProfiler" to perform gene ontology (GO) and Kyoto Encyclopedia of Genomes (KEGG) enrichment analyses. We also harvested the gene sets of the relevant pathways in the MSigDB database and calculated the correlation between PLBD1-related expression profiles and the pathways according to the GSEA algorithm. We assessed the therapeutic response to programmed cell death protein 1 (PD-1) and cytotoxic T-lymphocyte-associated protein 4 (CTLA4) immunotherapies in patients with PLBD1 high- and low-expression using the Cancer Immunome Atlas data (TCIA, https://tcia.at/home) 19.

### 2.6 Cell culture and transfection

The human glioma cell line, U87, were purchased from the Chinese Academy of Sciences cell bank (Shanghai, China). The glioma cell line was maintained in Dulbecco's Modified Eagle's Medium (HyClone, Logan, UT, USA), supplemented with 10% fetal bovine serum (Gibco, Carlsbad, CA, USA) and 1% penicillin/streptomycin (Gibco) at 37 °C with 5% CO_2_.

The vectors, sh-NC, sh-PLBD1, were transfected into U87 cells by Liposome 2000 transfection agent (Invitrogen, USA) according to the manufacturer's protocol. Sequences of shRNAs were: shRNA-1: GAGTCTACTATGCAACTGCAT; shRNA-2: GCAGAGGTCTACAACTTTGAT.

### 2.7 Quantitative Real-time PCR (qPCR)

As previously described 21, total RNA was extracted using the Mini-BEST Universal RNA Extraction kit (TaKaRa, Kyoto, Japan). Total RNA was reversely transcribed into first-strand cDNA using a Prime Script RT Master Mix reagent kit (TaKaRa), followed by qPCR assays with the SYBR Green Master Mix (TaKaRa) by PCR LightCycler480 (Roche Diagnostics, Basel, Switzerland). β-actin was used as an endogenous control.

### 2.8 Western blotting (WB)

As previously described 21, total proteins of U87 were isolated using a total cell protein extraction kit (KeyGen Biotechnology, Nanjing, China). Equal protein lysates were electrophoresed with SDS-PAGE, transferred to PVDF membranes, and blocked with 2% bovine serum albumin (KeyGen Biotechnology). The primary antibodies against PLBD1 (1:1000; Invitrogen) and GAPDH (1:2000, ProteinTech) were used to detect the expression at 4 °C overnight. After washing, secondary antibody (ProteinTech) was incubated. Bands were detected using a chemiluminescence ECL kit (Beyotime Biotechnology, Beijing, China) and quantified by Image J software (National Institutes of Health, Bethesda, MD, USA).

### 2.9 Immunohistochemistry (IHC)

As previously described 21, the paraffin-embedded tissue sections were deparaffinized and blocked. Then primary antibody against PLBD1 (1:100; Novus) were applied. After incubation in secondary antibodies, sections were treated with an immunohistochemical labelling kit (MaxVision Biotechnology, Fuzhou, China) and photographed with a light microscope (Olympus, Tokyo, Japan). The expression was evaluated by staining intensity and percentage of positive cells. The staining intensity was determined as follows: absent—0; weak—1; moderate—2; and strong—3. The percentage of positive cells was scored as follows: 0%, 0; 1-10%, 1; 11-50%, 2; 51-80%, 3; and 81-100%, 4. The immunohistochemical score was defined as the multiplication of both grading results (percentage of positive cells × staining intensities) and the positive expression was defined as a score≥4^22^, ^23^.

### 2.10 5-ethynyl-2′-deoxyuridine (Edu) assays

Edu assay was done to examine the activity of U87 by an Edu assay kit (Beyotime, Biotechnology). Briefly, U87 were co-cultured with Edu reagent for 2 hours. After fixed and permeabilized, U87 were counterstained. The images were taken by a laser scanning confocal microscope (Olympus) and the percentage of Edu-positive cells was calculated.

### 2.11 Transwell assay

As previously described, in the Transwell assay, glioma cells (10^4^ cells) under different conditions were placed in the upper chamber with a Matrigel filter (Corning) and DMEM containing 10% FBS was added to the lower chamber. After 24 hours of incubation, invading cells were fixed with 4% polymethanol and stained with crystal violet (Beyotime, Biotechnology). The stained cells were photographed and counted under a light microscope (Olympus).

### 2.12 Statistical analysis

Results are presented as the mean ± SD of at least three independent experiments. The two-tailed Student's t-test and one-way ANOVA were used to determine the statistical significance among different groups. The correlation between two groups were assessed by Pearson's correlation analysis. The survival difference was evaluated by log-rank test and Kaplan-Meier analysis. GraphPad was used for statistical analysis and P values < 0.05 were considered significant.

## 3. Results

### 3.1 Pan-cancer expression of PLBD1

**Figure 1:**
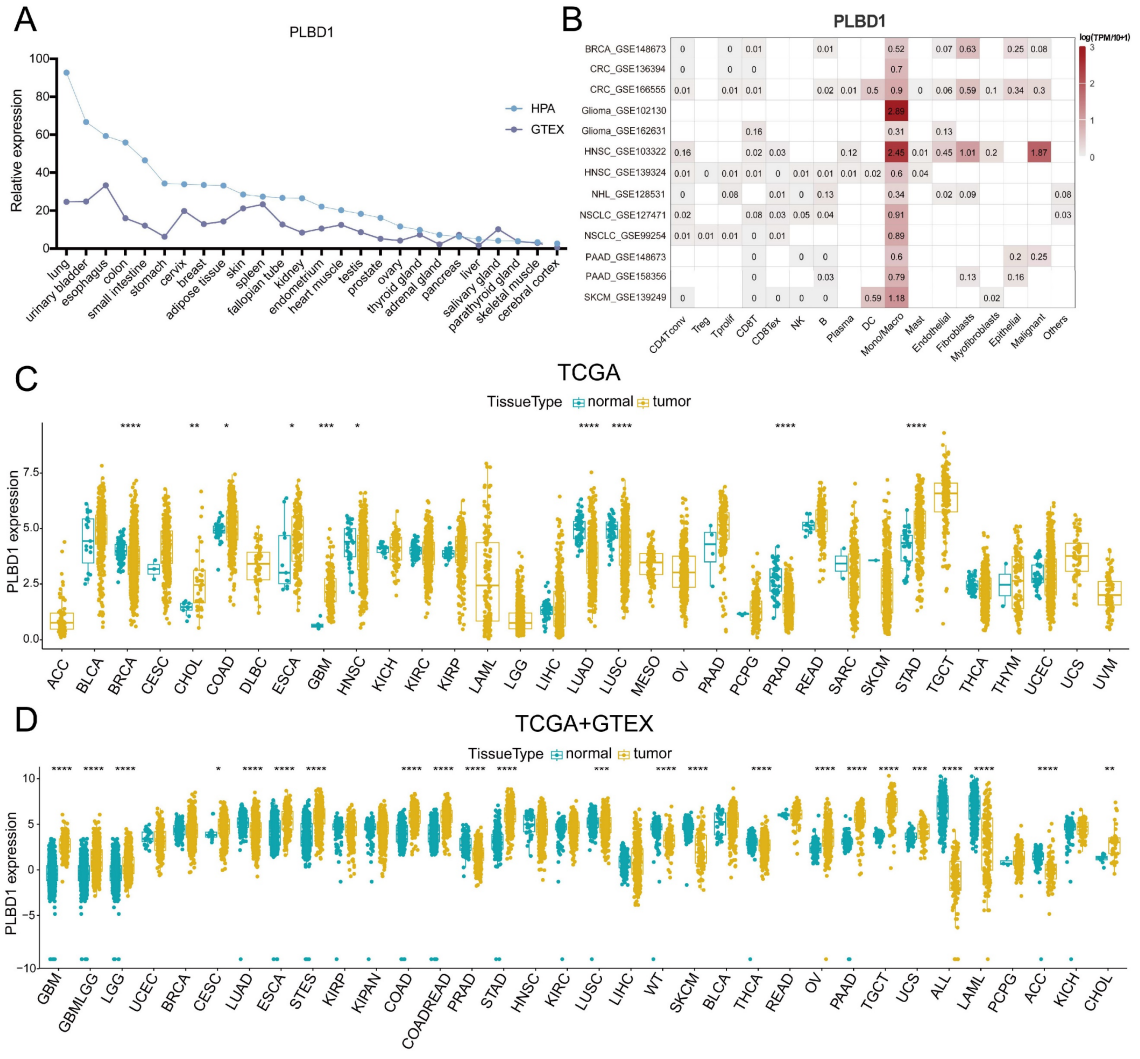
** The expression of PLBD1 in normal tissues, single-cell type, and tumor tissues.** (A) Expression level of PLBD1 in normal tissues (HPA+GTEx datasets); (B) The expression levels of PLBD1 in single cells (TISCH datasets); (C) PLBD1 expression levels in TCGA dataset; (D) The expression levels of PLBD1 between normal and tumor tissues (TCGA+GTEx datasets). *p < 0.05; **p < 0.01; ***p < 0.001; ****p < 0.0001.

To first characterize the expression landscape of PLBD1 in cancer, the GTEx and HPA database was applied to describe the levels of PLBD1 in normal tissues. The results revealed higher expression of PLBD1 in the lung, urinary bladder, colon, small intestine, esophagus, and other normal tissues (Figure 1A). We performed single-cell analysis of PLBD1 in multiple cancer single-cell datasets via the TISCH platform to understand the main cell types expressing PLBD1 in the tumor microenvironment. The results showed that PLBD1 was mainly expressed in macrophages and tumor cells (Figure 1B and Supplementary Figure 1A-B). In the GSE145281 dataset, PLBD1 was predominantly expressed in macrophages in the BLCA microenvironment (Supplementary Figure 1B). In the GSE125449 dataset, PLBD1 was highly expressed in tumor cells and macrophages (Supplementary Figure 1B). Moreover, analysis of TCGA dataset revealed high expression of PLBD1 in multiple cancers, including LGG and GBM (Figure 1C and Supplementary Figure 1C). Additionally, analysis of the integrated TCGA and GTEx data also showed the similar results (Figure 1D). Furthermore, we exhibited a significant correlation between PLBD1 expression and the pathological stages of some cancers, including LIHC, PAAD, READ and THCA (Supplementary Figure 2).

### 3.2 The prognostic value of PLBD1 in pan-cancer tissues

**Figure 2:**
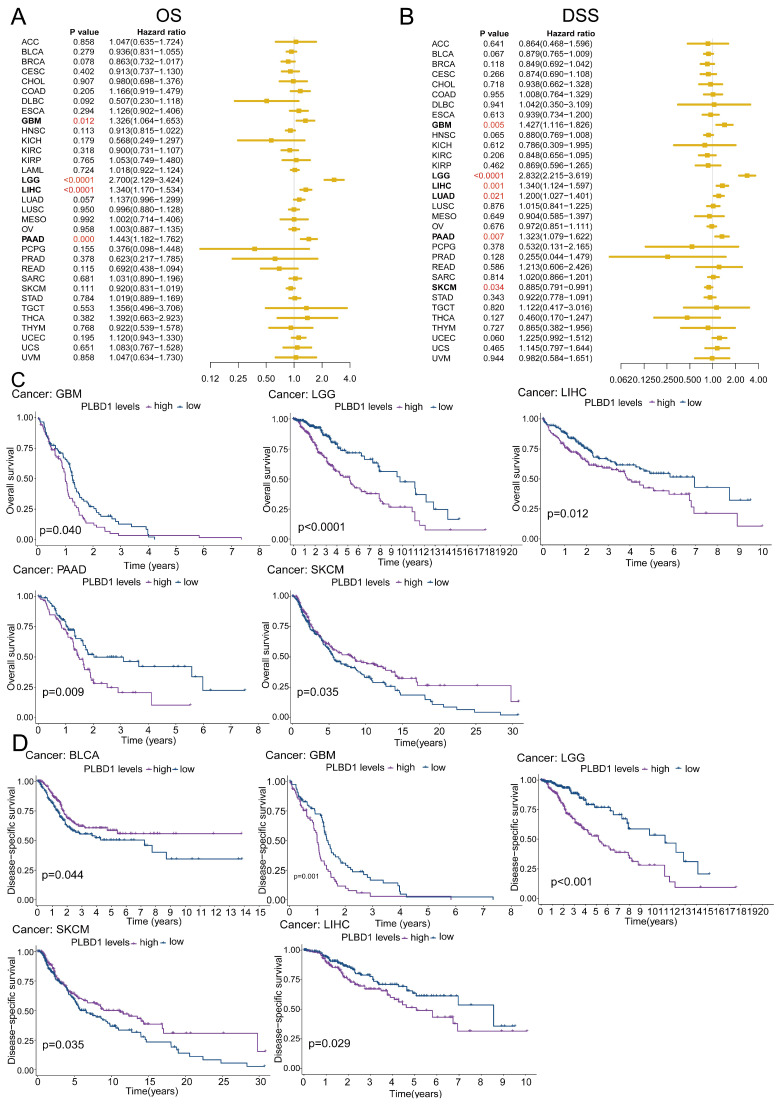
** The prognostic significance of PLBD1 in pan-cancer.** (A) Forest plot of association of PLBD1 expression and OS in pan-cancer; (B) Forest plot of association of PLBD1 expression and DSS in pan-cancer; (C) High expression of PLBD1 was significantly correlated with shorter OS in LGG, LIHC, GBM, PAAD and SKCM; (D) High expression of PLBD1 was significantly correlated with shorter DSS in BLCA, LGG, LIHC, GBM and SKCM.

To reveal the relationship between PLBD1 expression and the prognosis of patients, we conducted the prognostic analysis in pan-cancer. The results showed that high expression of PLBD1 was significantly correlated to shorter overall survival (OS), progression-free survival interval (PFI), disease-specific survival (DSS), and disease-free interval (DFI) in LIHC and PAAD (Figure 2A-D and Supplementary Figure 3A-D). Moreover, high expression of PLBD1 was significantly associated with shorter OS, PFI and DSS in LGG, GBM, LIHC and PAAD (Figure 2A-D and Supplementary Figure 3C-D). Furthermore, high expression of PLBD1 predicted shorter OS and DSS of patients with SKCM (Figure 2A-D), shorter DSS of patients with LUAD (Figure 2A-D), shorter DFI of patients with PRAD (Supplementary Figure 3A-B), shorter PFI of patients with KIRC (Supplementary Figure 3C-D).

### 3.3 The epigenetic variations of PLBD1 in pan-cancer

**Figure 3:**
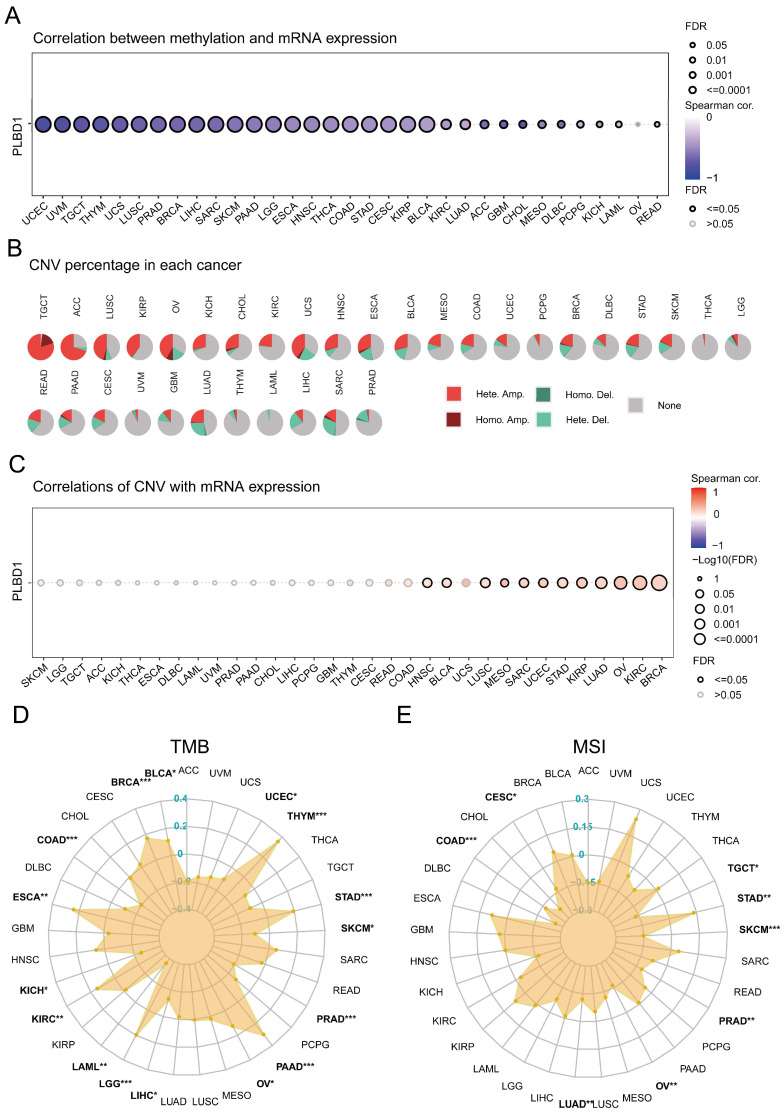
** The epigenetic landscape of PLBD1 in pan-cancer.** (A) The relationship between methylation and expression of PLBD1 in pan-cancer; (B) Copy number variation levels of PLBD1 in pan-cancer; (C) The relationship between copy number variation levels and expression of PLBD1 in pan-cancer; (D) Relationship between PLBD1 and tumor mutational burden in pan-cancer; (E) Relationship between PLBD1 and microsatellite instability in pan-cancer. *p < 0.05; **p < 0.01; ***p < 0.001.

Considering the abnormal expression of PLBD1 in pan-cancer, we further showed the genetic alteration status of PLBD1 across pan-cancer samples in TCGA datasets. In-depth, as shown in Supplementary Figure 4A-B, the highest alteration frequency of PLBD1 (16%) appeared in SKCM and UCEC tumors with “Missense_Mutation” and “Nonsense_Mutation” as the primary types. Moreover, we explored the association between the expression and methylation levels of PLBD1. The results showed that the methylation level of PLBD1 was negatively correlated with its mRNA expression to varying degrees in pan-cancer (Figure 3A). Furthermore, prognostic analyses showed that in BLCA, hypermethylation levels of PLBD1 were significantly associated with shorter survival time, whereas hypomethylation levels of PLBD1 were significantly associated with poor prognosis in LGG, LIHC and PAAD (Supplementary Figure 4C).

In addition, PLBD1 has highly heterogeneous CNVs in different tumors, of which we analyzed homozygous and heterozygous deletions and amplifications. The results showed that heterozygous amplification was prevailing in TGCT and ACC patients, while heterozygous deletions were observed in SARC, LUAD, and LIHC patients (Figure 3B). The correlation analysis revealed that the CNVs of PLBD1 was positively correlated with its mRNA expression in multiple cancers including GBM (Figure 3C). TMB and MSI were closely involved in clinical management of tumors and tumor markers. In tumors such as BLCA, ESCA, LGG, LIHC, and PRAD, the expression of PLBD1 was significantly related to TMB (Figure 3D); in tumors such as CESC, LUAD, PRAD, and STAD, the expression of PLBD1 was significantly associated with MSI (Figure 3E).

### 3.4 The relationship between PLBD1 and immunological features and immunotherapy response in pan-cancer

**Figure 4:**
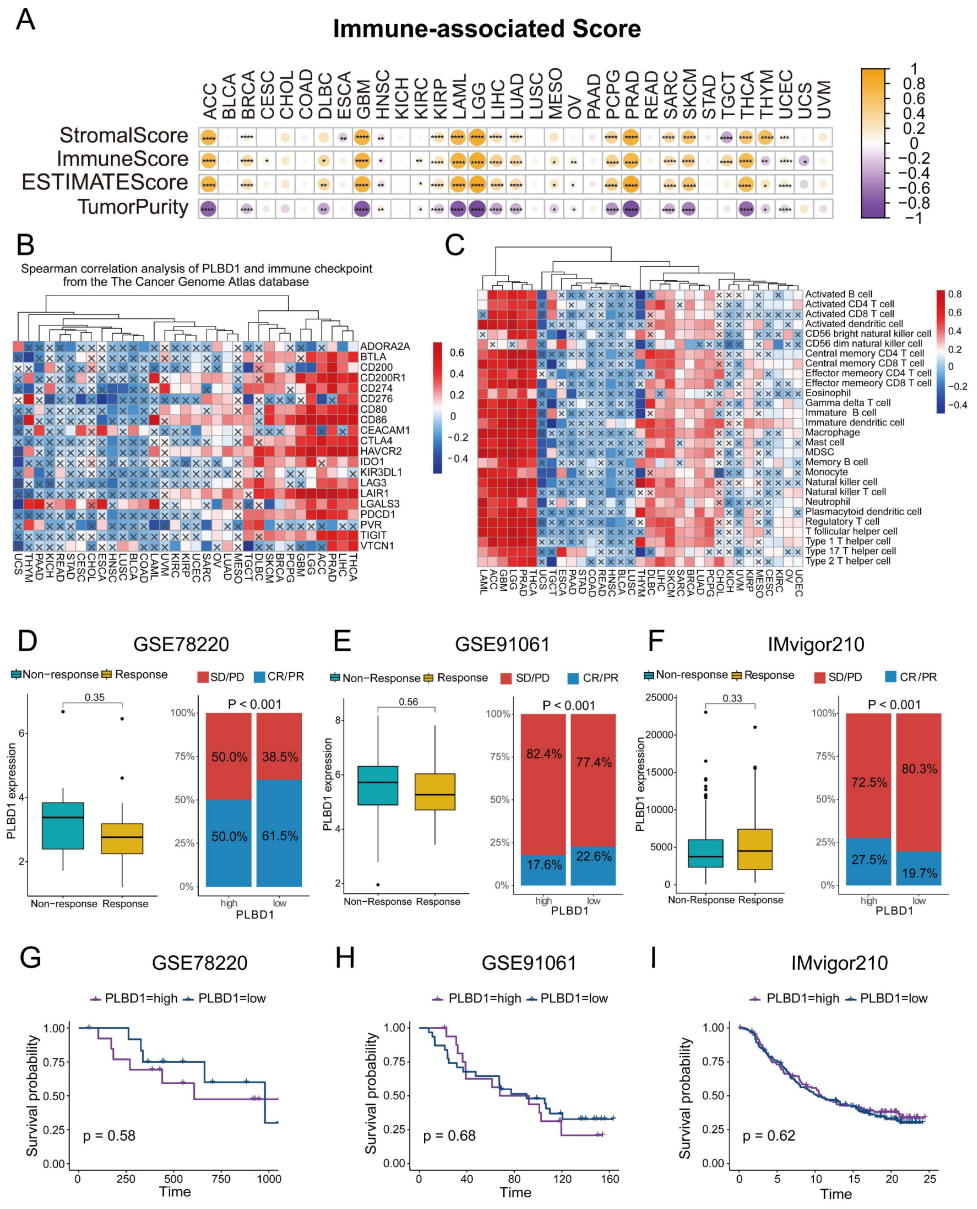
** The relationship between PLBD1 expression and immunological characteristics of pan-cancer tissues.** (A) The relationship between PLBD1 and immune score, stromal score, tumor purity and ESTIMATE scores in pan-cancer; (B) The correlation between PLBD1 and immune checkpoint from TCGA dataset; (C) The correlation between PLBD1 and immune cells infiltration with ssGSEA from TCGA dataset; (D, G) Effects of PLBD1 expression on anti-PD-L1 treatment response and OS in GSE78220 cohorts; (E, H) Effects of PLBD1 expression on anti-PD-L1 treatment response and OS in GSE91061 cohorts; (F, I) Effects of PLBD1 expression on anti-PD-L1 treatment response and OS in IMvigor210 cohorts. *p < 0.05; **p < 0.01; ***p < 0.001; ****p < 0.0001.

To investigate the association between PLBD1 and immunological features, we calculated the immune score, stromal score, and tumor purity, and analyzed their correlation with PLBD1 expression. The results indicated that PLBD1 has a strong positive correlation with immune score and stromal score, and has a negative correlation with tumor purity in several cancers, including ACC, LGG, GBM, PRAD, etc (Figure 4A). Moreover, PLBD1 has a strong positive correlation with immune checkpoints like PDCD1 and CTLA-4 in multiple tumors including THCA, LIHC, ACC, LGG and GBM, etc (Figure 4B). Furthermore, we used 7 algorithms including ssGSEA, CIBERSORT, CIBERSORT-ABS, EPIC, XCELL, TIMER and MCPCOUNTER to assess the association of PLBD1 with immune cells in tumor microenvironment. The results showed that high PLBD1 expression was strongly positive related to various levels of infiltrated immune cells, especially immunosuppressive cells such as Myeloid-derived suppressor cells (MDSCs) and M2 macrophages in LGG, LIHC, GBM, THCA, etc (Figure 4C and Supplementary Figure 5A-F). Neoantigens, a group of tumor-specific antigens generated by tumor cell mutations, have the potential to be valuable targets for tumor immunotherapy 24, 25. The expression of neoantigens showed a close correlation with PLBD1 levels in KICH, THYM, COAD, LGG, BRCA, THCA, SARC, GBM and PCPG (Supplementary Figure 5G).

Next, we investigated the effect of PLBD1 on the response to immunotherapy using three immunotherapy cohorts of oncology patients. In GSE78220 and GSE91061, PLBD1 expression was higher in patients who did not respond to immunotherapy than in those who did, although not statistically significant (Figure 4D-E). In GSE78220 and GSE91061, patients with low PLBD1 expression were much more possible to achieve complete or partial response (Figure 4 D-E). In GSE78220, survival time was decreased in patients with high PLBD1 expression, whereas in GSE91061 and IMvigor210, there was no difference in survival time between patients with high and low PLBD1 expression (Figure 4G-I).

### 3.5 The expression and prognostic value of PLBD1 in glioma datasets and tissues

**Figure 5:**
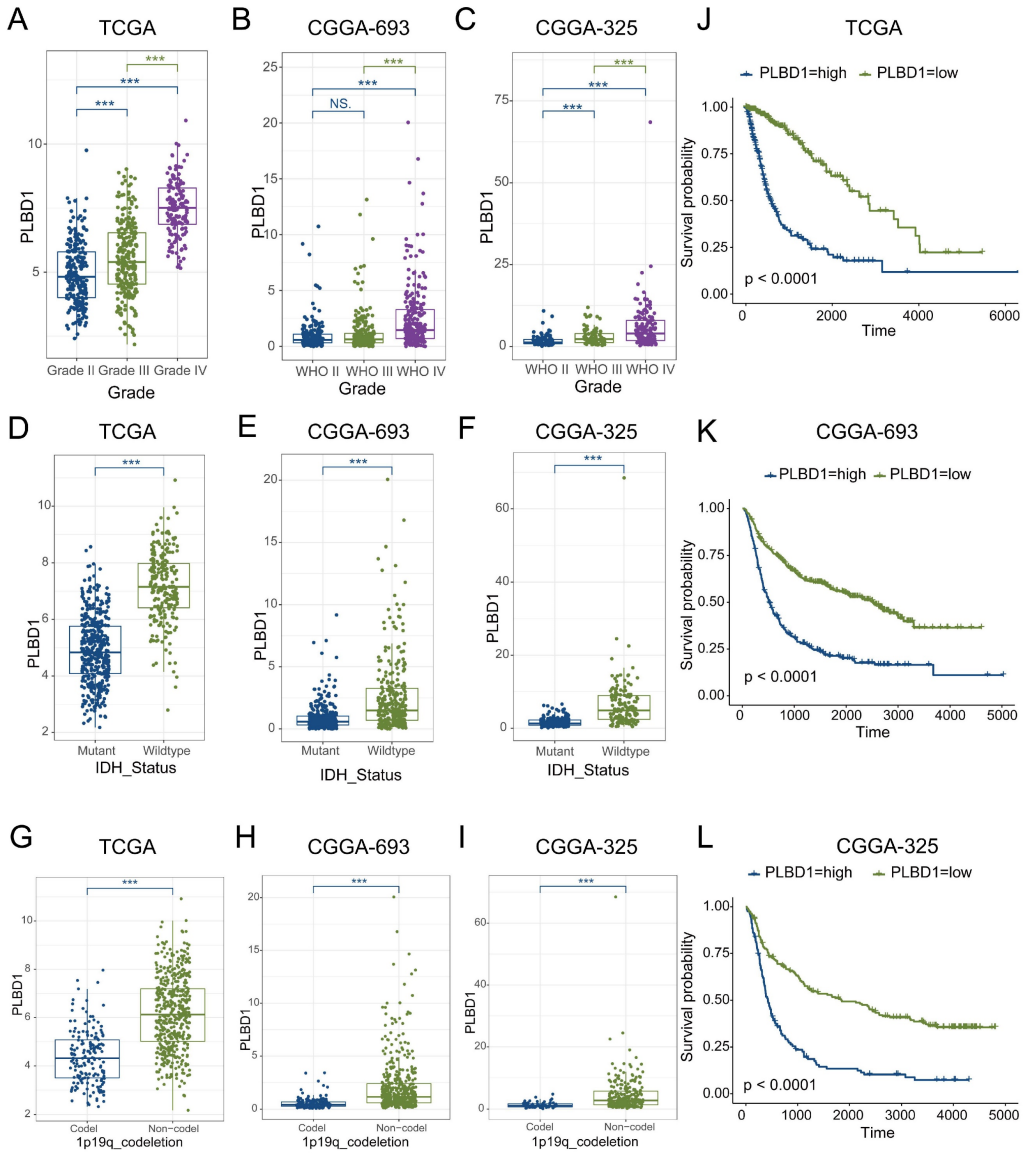
** The expression and prognostic value of PLBD1 in glioma datasets.** (A-C) The mRNA expressions of PLBD1 are shown according to WHO grades in TCGA (A), CGGA-693 (B) and CGGA-325 (C) datasets; (D-F) The mRNA expressions of PLBD1 are shown according to IDH status in TCGA (D), CGGA-693 (E) and CGGA-325 (F) datasets; (G-I) The mRNA expressions of PLBD1 are shown according to 1p19q_codeletion status in TCGA (G), CGGA-693 (H) and CGGA-325 (I) datasets; (J-L) The survival analysis of PLBD1 expression in TCGA (J), CGGA-693 (K) and CGGA-325 (L) datasets. ***p < 0.001; NS, No Significance.

**Figure 6:**
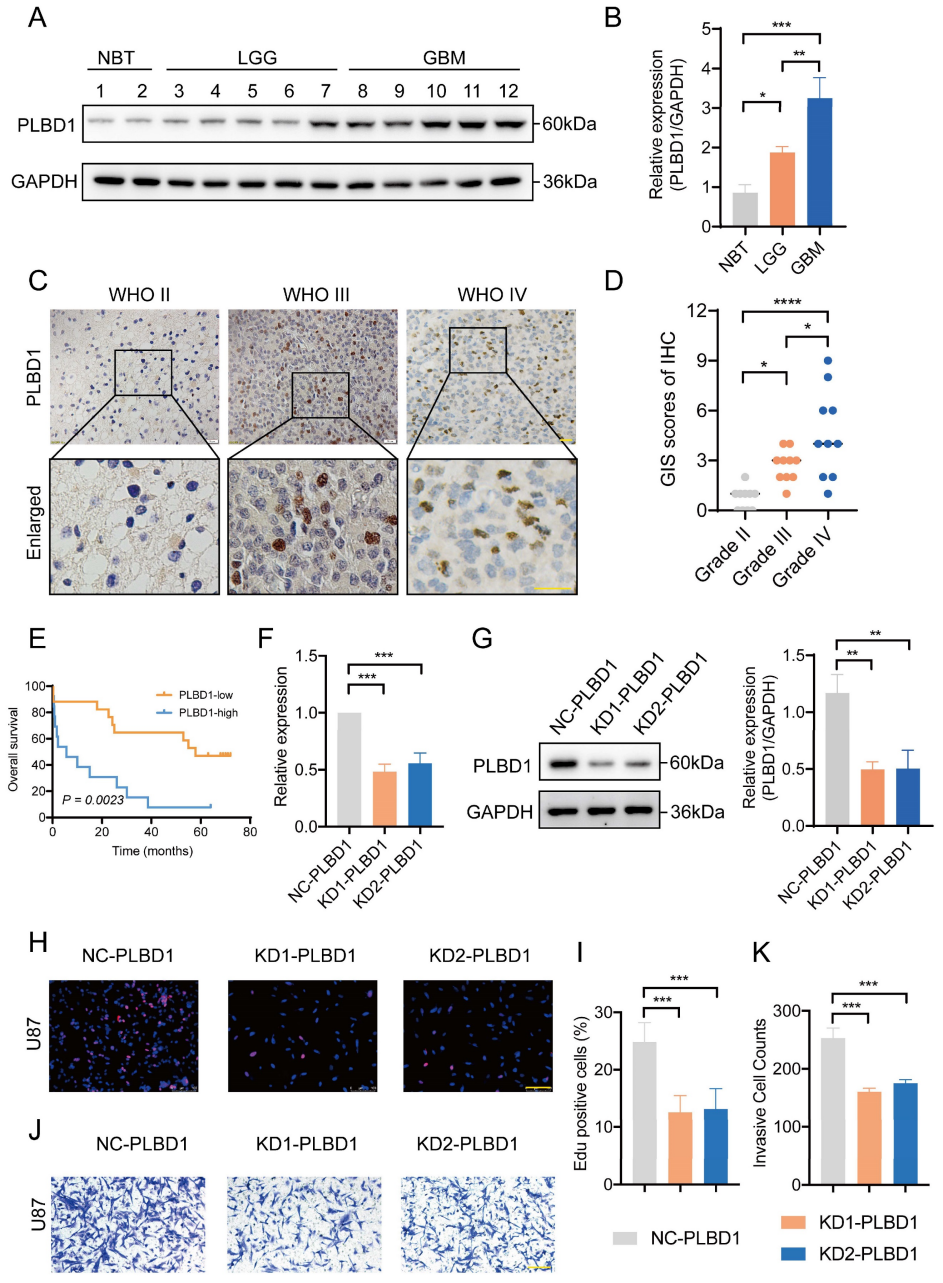
** The expression of PLBD1 in glioma tissues from our cohort and the effect of PLBD1 knockdown on the proliferation and invasion of glioma cells.** (A-B) The expression of PLBD1 in normal brain tissues and WHO II-IV glioma tissues, as detected by western blots; (C-D) The expression of PLBD1 in WHO II-IV glioma tissues, as detected and analyzed by IHC; scale bar = 20μm; (E) The prognostic value of PLBD1 expression in glioma patients from our cohort; (F) PLBD1 is silenced in U87 glioma cells by two different shRNAs. The transfected efficacy is detected by qPCR; (G) PLBD1 is silenced in U87 glioma cells by two different shRNAs. The transfected efficacy is detected by western blots. GAPDH is used as a loading control; (H-I) The Edu assay show the proliferation of U87 glioma cells after PLBD1 is silenced. Scale bar = 100μm; (J-K) The Transwell assay show the invasion of U87 glioma cells after PLBD1 is silenced. Scale bar = 100μm. *p < 0.05; **p < 0.01; ***p < 0.001; ****p < 0.0001.

Considering the expression of PLBD1 with in LGG and GBM and its correlation with prognosis and immune characteristics, we further evaluated the role and clinical value of PLBD1 in gliomas. We then combined the expression and prognosis matrix of TCGA-LGG and TCGA-GBM and assessed the expression, prognostic significance and the functional enrichment of PLBD1 in glioma. The results showed that PLBD1 was significantly enriched in high-grade glioma (especially GBM), non-1p/19q deletion status and IDH wild-type in TCGA (Figure 5A, D, G), CGGA-693 (Figure 5B, E, H) and CGGA-325 datasets (Figure 5C, F, I). PLBD1 also highly expressed in non-O-methylguanine-DNA-methyltransferase (MGMT) promoter methylated samples in CGGA-325 and CGGA-693 datasets (Supplementary Figure 6A-B). These results indicated that PLBD1 was highly enriched in gliomas with more malignant behavior. The prognostic results showed that glioma patients with high PLBD1 expression had a significantly shorter survival time than those with low PLBD1 expression in TCGA (Figure 5J), CGGA-693 (Figure 5K) and CGGA-325 datasets (Figure 5L). In CGGA-693 dataset, we also found that PLBD1 high expression predicted worse prognosis in WHO II, III and GBM samples, respectively (Supplementary Figure 6C). To validate the expression of PLBD1 in glioma datasets, we further examined the PLBD1 expression in our patients' samples by western blots and IHC. The results showed that the expression of PLBD1 was absent in normal brain tissues and positively correlated with glioma grade (Figure 6A-B). PLBD1 was also highest expressed in the nuclei of GBM (Figure 6C-D). Patients with high PLBD1 expression had worse prognosis in our cohort (Figure 6E). These results indicated that PLBD1 was confirmed to be upregulated in high-grade glioma and be related to the clinical prognosis of glioma.

### 3.6 Knockdown of PLBD1 expression reduced the proliferation and invasion of glioma cells *in vitro*

We first transfected U87 glioma cells with two shRNA knockdown vectors and conducted RT-PCR and western blot analysis. The results showed that the mRNA (Figure 6E) and protein expression (Figure 6F-G) of PLBD1 in the knockdown group were significantly lower than in the control group. The knockdown efficiency was similar between two knockdown groups. Furthermore, we conducted EdU assay to evaluate the proliferation of glioma cells and performed Transwell to assess the invasion of glioma cells. The EdU results showed that compared to the control group, the ratio of proliferating glioma cells in the PLBD1 knockdown groups were significantly reduced (Figure 6H-I). Moreover, the Transwell results indicated that compared to the control group, the number of invaded glioma cells in the PLBD1 knockdown groups were significantly decreased (Figure 6J-K). These results suggested that knockdown of PLBD1 expression could inhibit the proliferation and invasion ability of glioma cells *in vitro*.

### 3.7 Functional enrichments, immunological features and immunotherapy response of PLBD1 in glioma

**Figure 7:**
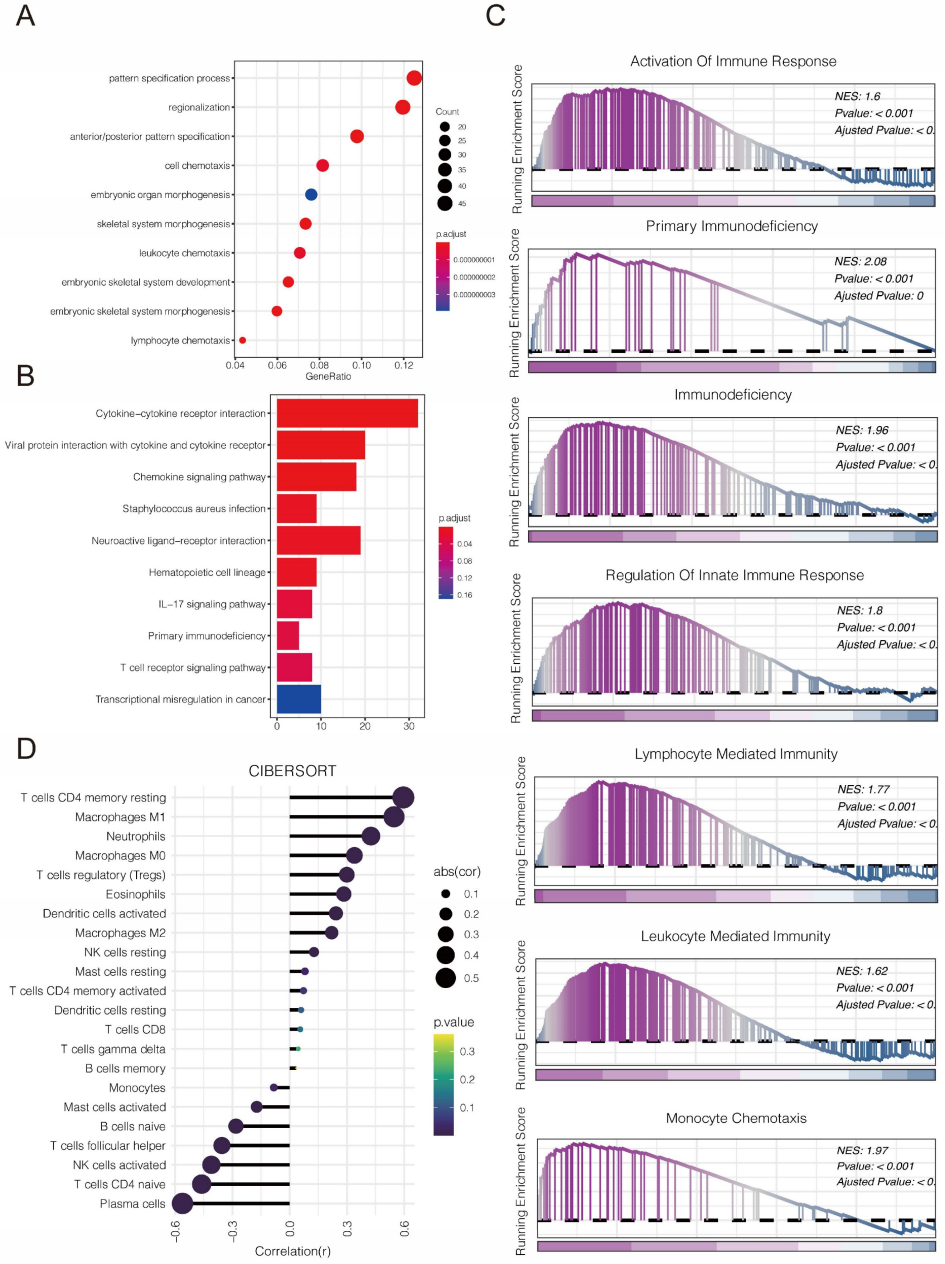
** The relationship between PLBD1 expression, functional enrichments, and immunological features in glioma.** (A) GO functional enrichment analysis between PLBD1 high- and low-expression group; (B) KEGG functional enrichment analysis between PLBD1 high- and low-expression group; (C) GSEA functional enrichment analysis between PLBD1 high- and low-expression group; (D) The correlation between PLBD1 expression and immune cells infiltration with CIBERSORT in TCGA glioma dataset.

We first explored the function of PLBD1 in glioma in TCGA dataset. GO analysis revealed that the differentially expressed genes were significantly enriched in immune-related biological process, such as pattern specification process, cell chemotaxis, leukocyte chemotaxis and lymphocyte chemotaxis (Figure 7A). KEGG enrichment analysis showed that the differentially expressed genes were enriched in chemokine signaling pathway and cytokine-cytokine receptor interactions (Figure 7B), while GSEA analysis indicated the enrichments in immune activities, such as activation of immune response, lymphocyte mediated immunity, leukocyte mediated immunity and monocyte chemotaxis (Figure 7C).

**Figure 8:**
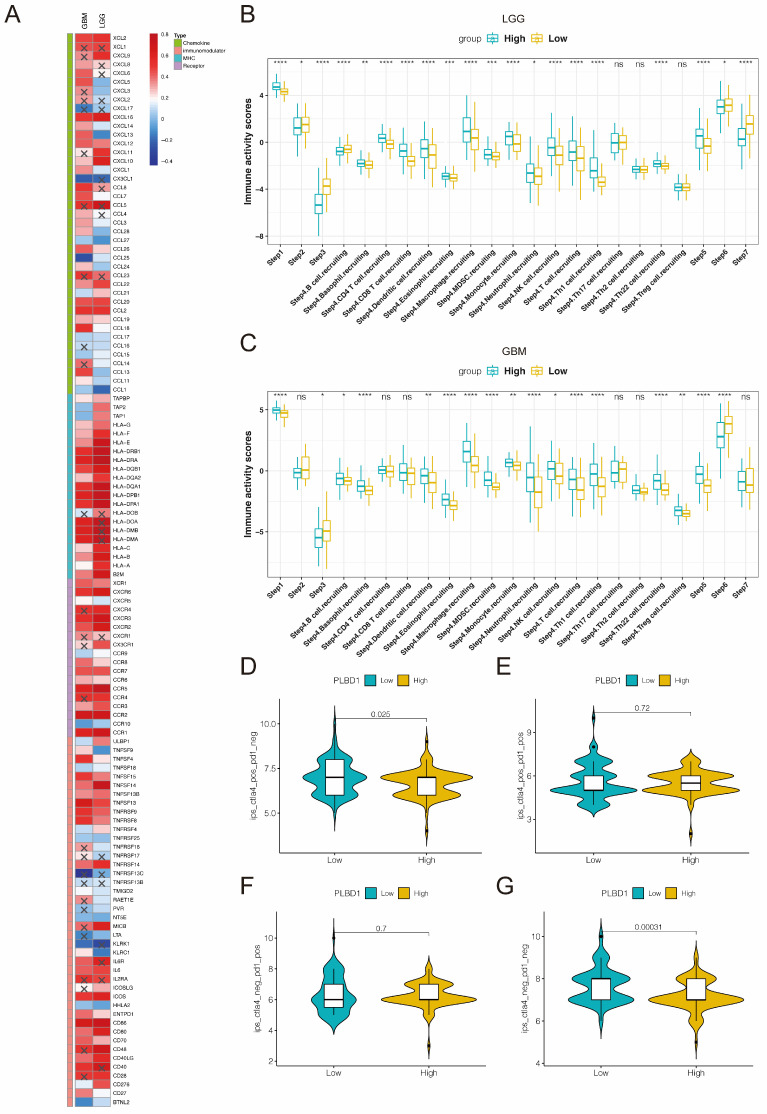
** The correlation between PLBD1 expression and immune modulators, cancer immunity cycle and immunotherapy response.** (A) The association of PLBD1 with immune modulators (chemokines, receptors, MHC and immunostimulants) in glioma; (B-C) Differences in the various steps of the cancer immunity cycle between PLBD1 high- and low-expression groups in LGG (B) and GBM (C); (D-G) Correlation between PLBD1 expression and immunotherapy response (Immunophenoscore) in GBM from TCIA website. *p < 0.05; **p < 0.01; ***p < 0.001; ****p < 0.0001.

Considering the key role of PLBD1 in tumor immunity, we then evaluated the correlation between PLBD1 and immune cells, immune modulators, immune process and immunotherapy response. The CIBERSORT showed that PLBD1 in the glioma had a significant correlation with various immune cells such as macrophages, neutrophils and Tregs (Figure 7D). Moreover, chemokines like CCL2, CCL22, etc. were key factors of monocytes/macrophages infiltration. The expression of these chemokines was significant higher in the PLBD1 high-expression group than in the PLBD1 low-expression group. In addition, the expression of major histocompatibility complex (MHC) molecules was upregulated in glioma in PLBD1 high-expression group, suggesting the increasing antigen presentation abilities (Figure 8A). In addition, we also noted that the PLBD1 high expression group had a more robust anti-tumor immune status in most steps of the immune cycle, including release of cancer cell antigen (step 1), priming and activation (step 3), trafficking of immune cells to tumors (step 4), infiltration of immune cells into tumors (step 5), and killing of the cancer cells (step 7) (Figure 8B-C).The enhanced immune status of the PLBD1 high expression group may further augment the immune cell function in the LGG and GBM microenvironments. We also found that PLBD1 expression was adversely linked to T-cell recognition of cancer cells (step 6). High expression of PLBD1 may reduce T-cell receptor recognition and further exacerbate immune escape. Due to the difference in immune status between the PLBD1 high and low expression groups, it is suggested that the response to immunotherapy may be different between the two groups. We found that the group with low PLBD1 expression benefited more from immune checkpoint (PD-1 and CTLA-4) blockade therapy than the group with high PLBD1 expression (Figure 8D-G).

## 4. Discussion

Transcription factors, as key factors of transcriptional regulation, regulate the transcription and expression of genes, thus affecting the physiological functions of cells and the occurrence and development of tumors 26, 27. Dysregulation of transcription is one of the important hallmarks of tumor development, and the discovery of key proteins for targeting the transcriptional regulation involved in cancer therapeutics is a popular strategy in the research and development of anti-tumor drugs 28. The OCT-4, SOX-2, NANOG, KLF4, MYC, Wnt, Notch signaling pathways, as well as the JAK-STAT, PI3K and NF-kβ signaling pathways, are currently the major transcription factor signaling pathways 29, 30. Among them, early clinical trials of inhibitors of OCT-4, SOX-2, NANOG and Notch pathways have made significant progress 31. However, they are not currently available for effective use in the clinic. Therefore, there is an urgent need to find new transcription factors serving as biomarkers and therapeutic targets for tumor progression.

PLBD1 is a transcription factor that regulates phospholipid metabolism. Previous literature suggests a link between PLBD1 and cardiovascular diseases. The study suggests that PLBD1 RNA expression in peripheral blood may be a novel and independent predictor of left ventricular insufficiency after acute myocardial infarction 9, and PLBD1 also serve as a characteristic transcription factor in peripheral blood of ischemic stroke patients 10. In addition, PLBD1 may be used as a prognostic biomarker for psoriasis and as a target for the treatment of patients with psoriasis 8. However, little is known about the role of PLBD1 in tumors. Previous study found that PLBD1 was valuable as a diagnostic and prognostic marker for pancreatic ductal adenocarcinoma 12. Although several studies have suggested that risk models constructed from multiple genes, including PLBD1, may predict the prognosis of glioma patients and reflect the immunological characteristics of gliomas 13, 14, the relationship between PLBD1 and a variety of tumors, especially glioma, has not yet been thoroughly clarified.

In this study, we performed a complete bioinformatics analysis using patient data from different repositories to determine the functional role of PLBD1 in a wide range of cancers. Among the 24 cancer types with normal tissues, 10 cancer types had statistically significant expression of PLBD1 in normal tissues and tumors. After integrating GTEX data, there were 23 cancer types with statistically significant PLBD1 expression in normal tissues and tumors. We found that the abundance and trend of PLBD1 expression in multiple tumors were not consistent, which may be related to tumor specificity and tumor microenvironment. In gliomas, PLBD1 was expressed to a significantly higher extent in GBM than in LGG, and the expression of PLBD1 was higher in patients with IDH mutations than in patients with IDH wild-type, which is thought to be the key to better prognosis 32. Survival analysis using TCGA and CGGA data revealed that high PLBD1 expression was associated with poor prognosis. These results suggest that PLBD1 is a potential prognostic biomarker for various cancers, especially gliomas.

In recent years, immunotherapy of tumors has shown remarkable therapeutic efficacy mainly by revitalizing the ability of the immune system to recognize tumor antigens and to clear tumor cells, but this is only possible with satisfactory efficacy in a small minority of tumors 33, 34. Immune cells infiltrated in the tumor microenvironment play an important role in the malignant progression of tumors, not only by providing a suitable immunosuppressive microenvironment for the survival of tumor cells, but also by being associated with tumor immunosurveillance and response to immunotherapy 35.

In view of this, we focused on exploring the impact of PLBD1 on tumor immune profiles. We found that PLBD1 was positively correlated with immune scores and stromal scores and negatively correlated with tumor purity in multiple cancers. Moreover, PLBD1 was closely associated with the expression of multiple immune checkpoints and the number of infiltrating immune cells, and had a relationship with the clinical response of immunotherapy patients. In addition, TMB is a prospective biomarker for pan-cancer immunotherapy prediction 36, 37. MSI can forecast sensitivity to treatment with immune checkpoint inhibitors, especially PD-1/PD-L1 inhibitors 38. Our analysis showed that PLBD1 expression was associated with TMB in 16 cancer types and MSI in 8 cancer types. The results suggest that PLBD1 modulates tumor immunity in multiple cancer types. Therefore, PLBD1 may be a potential biomarker for predicting the response to immunotherapy in patients with malignant tumors.

We identified a possible important role for PLBD1 in LGG and GBM during our analysis, and we examined PLBD1 expression in clinical samples collected from our research center. It was found that, consistent with database results, PLBD1 expression increased with the grade of glioma and was highest in GBM. These results suggest that PLBD1 may regulate the malignant progression of gliomas. We further knocked down PLBD1 in glioma cells and found that the proliferation and invasive ability of glioma cells were significantly reduced. GO, KEGG and GSEA analysis revealed that PLBD1-associated differential genes were significantly enriched in immune-related biological processes and signaling pathways. Moreover, PLBD1 expression was closely associated with the proportion of immune cells, especially macrophages, and a variety of chemokines, MHC and other immunomodulatory molecules. Complex and dynamic interactions between secreted cytokines, chemokines, growth factors and their receptors mediate the immunosuppressive tumor microenvironment, leading to tumor progression and treatment resistance, including GBM 39. Antigen processing and presentation is a complex process that can be exploited by tumors to evade immune recognition 40. PLBD1 may be involved in the above process. We also observed a significant positive correlation between PLBD1 and several steps of the cancer immune cycle in gliomas. For example, there was a significant increase in macrophage and monocyte infiltration in the PLBD1 high-expression group, which may be due to a marked increase in macrophage recruitment. In addition, PLBD1 expression was significantly negatively correlated with T cell recognition of cancer cells (step 6), which may be due to significantly increased expression of several inhibitory immune checkpoints in the PLBD1 high expression group.

In summary, although we have performed cellular experiments *in vitro* to investigate the biological function of PLBD1 in gliomas, further biological assays *in vivo* are needed to confirm our findings and to explore the mechanism of action in depth.

## 5. Conclusion

Our findings indicate that PLBD1 is significantly highly expressed in a variety of cancers and significantly correlates with the prognosis of patients. In some cancer types, PLBD1 levels were associated with TMB, MSI, immune cell infiltration, expression of immunosuppressive molecules, and immunotherapeutic response. PLBD1 expression was strongly correlated with the prognosis of gliomas, glioma cell proliferation and invasion, which was preliminarily validated in cellular models. PLBD1 expression was also significantly correlated with immune cell infiltration of gliomas, immune cycling, and immunotherapy. In brief, PLBD1 is considered a promising biomarker for predicting the prognosis of gliomas, which may inform the achievement of more precise individualized immunotherapy in the future.

## Supplementary Material

Supplementary figures and table.
