# Supplementary material for: The prognostic and immune significance of PLBD1 in pan-cancer and its roles in proliferation and invasion of glioma
Source: J Cancer. 2024 May 20;15(12):3857–72. doi: 10.7150/jca.96365 (PMC11190780; doi:10.7150/jca.96365)
Supplement: Supplementary file 1 — Supplementary figures and table. [file jcav15p3857s1.pdf]

Supplementary Table 1. Basic information of patients in our cohort

**Characteristic**	**Tangdu cohort**
**Total**	40
**Age**	
>=42	25
<42	15
**Gender**	
Male	27
Female	13
**Grade**	
II	12
III	13
IV	15
**Histology**	
Astrocytoma	14
Oligodendroglioma	11
Glioblastoma	15
**IDH mutation status**	
Yes	18
No	22
**1p19q codeletion status**	
Yes	19
No	21
**MGMTp methylation status**	
Yes	12
No	28

Supplementary Figures

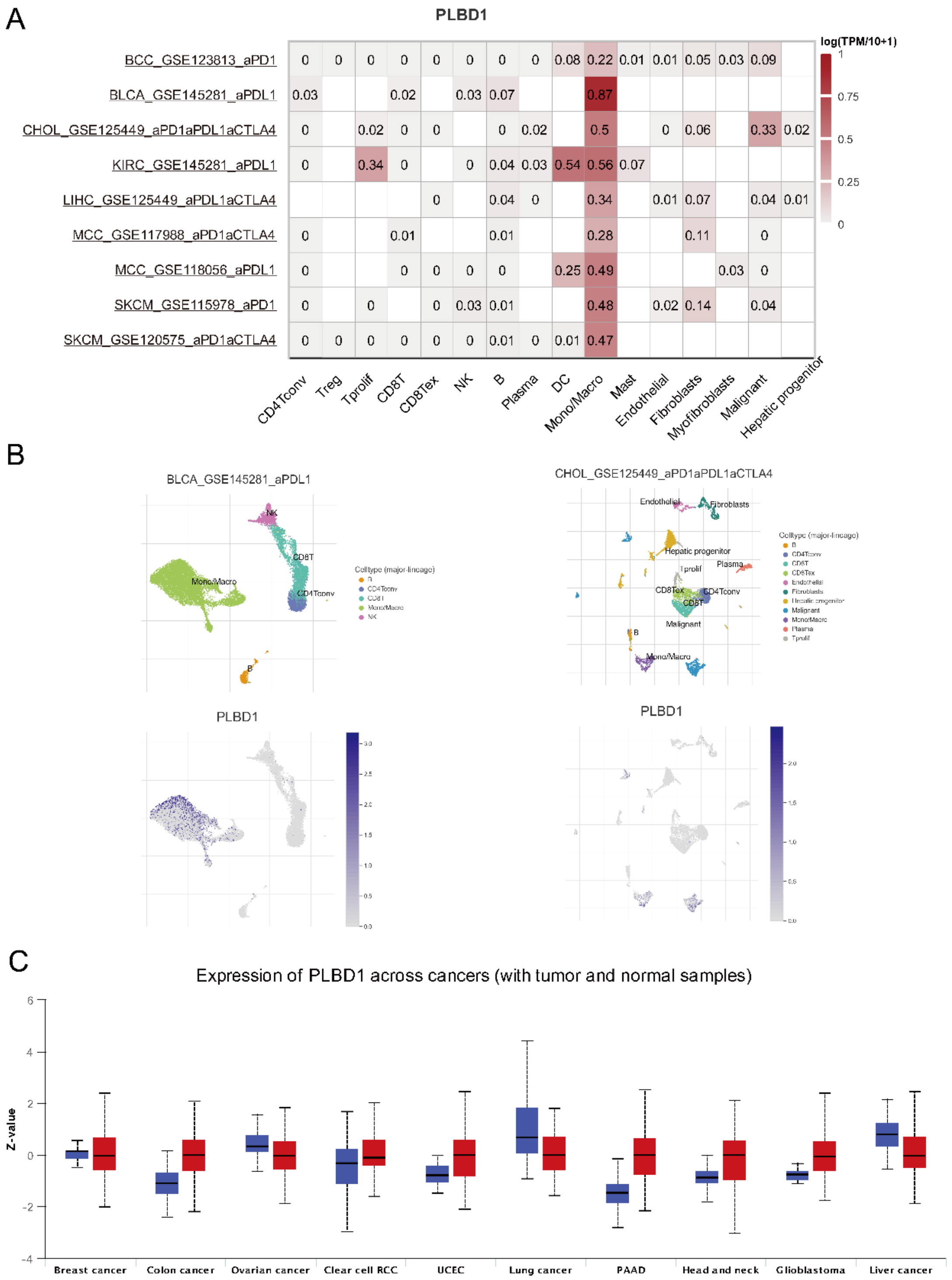

Supplementary Figure 1. The expression and location of PLBD1 in normal tissuesand tumor tissues. (A) The expression levels of PLBD1 in single cells (TISCH datasets); (B) The location of PLBD1 in single cells from GSE145281 and GSE125449 (TISCH datasets); (C) PLBD1 expression levels in TCGA dataset from CPTAC.

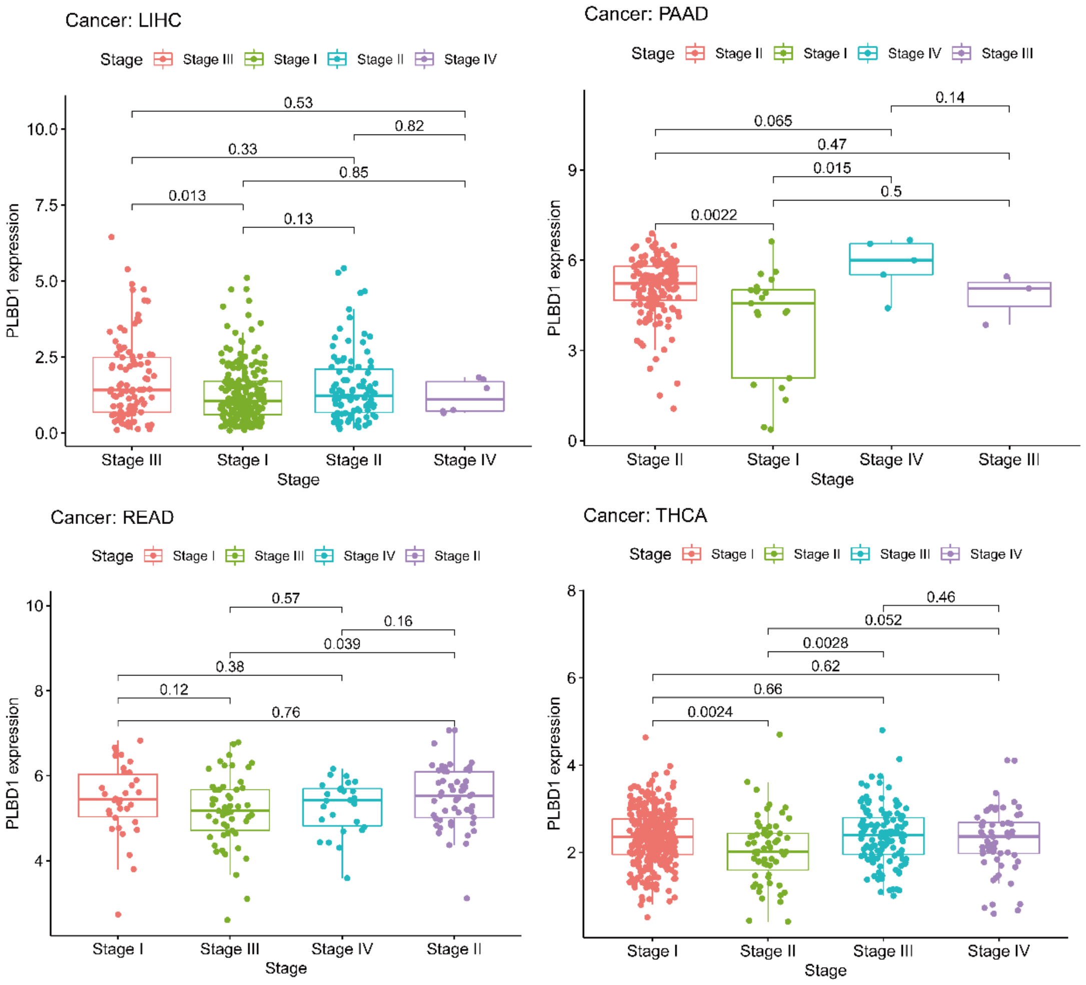

Supplementary Figure 2. The relationship between PLBD1 expression and tumor stages in several cancers.

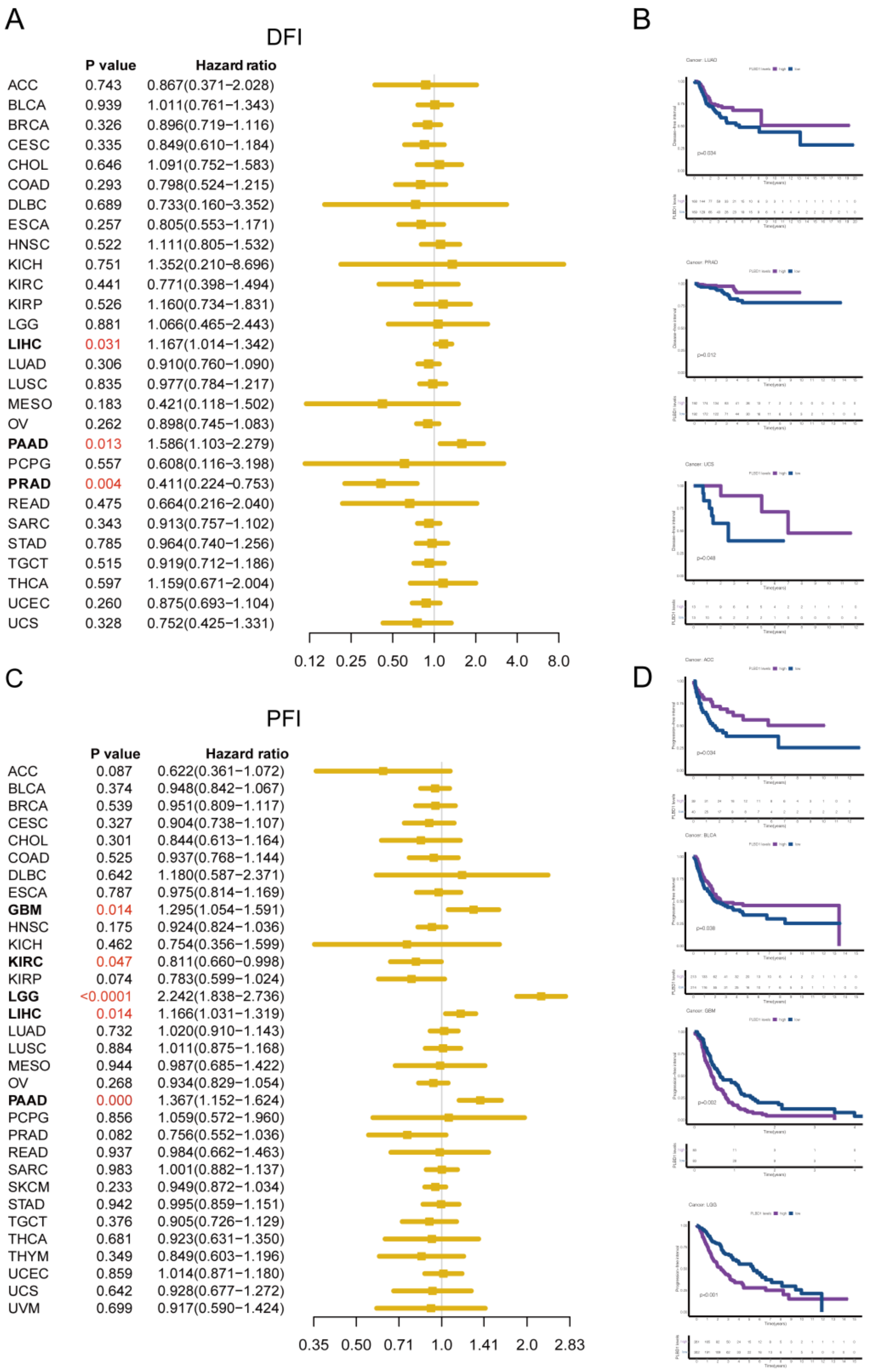

Supplementary Figure 3. The prognostic significance of PLBD1 in pan-cancer. (A) Forest plot of association of PLBD1 expression and DFI in pan-cancer; (B) High expression of PLBD1 was significantly correlated with shorter DFI in LUAD, PRAD and UCS; (C) Forest plot of association of PLBD1 expression and PFI in pan-cancer; (D) High expression of PLBD1 was significantly correlated with shorter PFI in ACC, BLCA, LGG, and GBM.

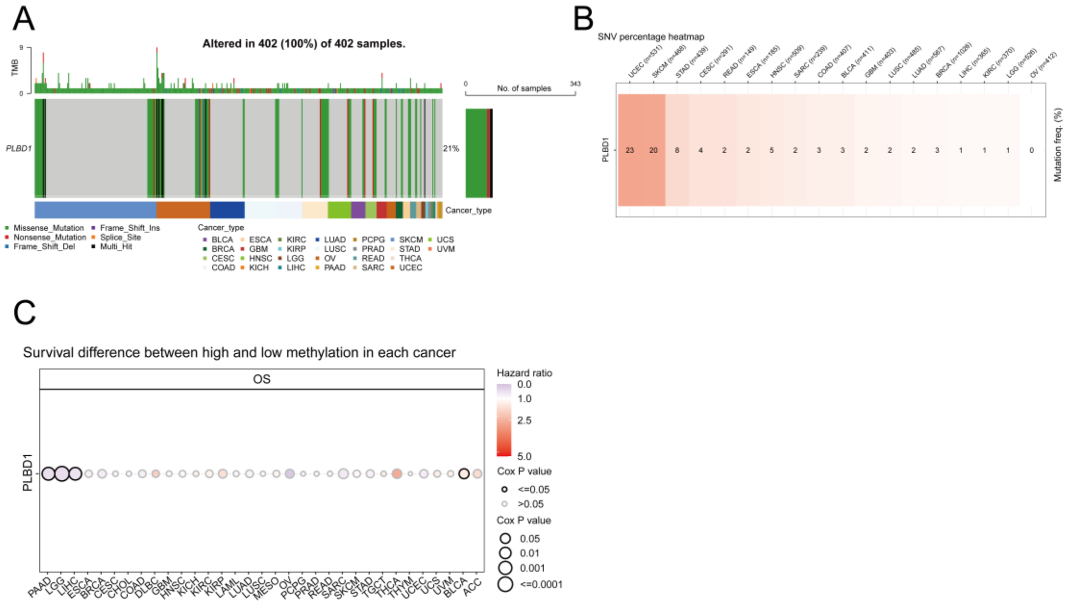

Supplementary Figure 4. The mutation frequency of PLBD1 in pan-cancer. (A) PLBD1 alteration frequency analysis in the pan-cancer study; (B) The mutation frequency of PLBD1 in pan-cancer; (C) The relationship between methylation of PLBD1 and prognosis in pan-cancer.

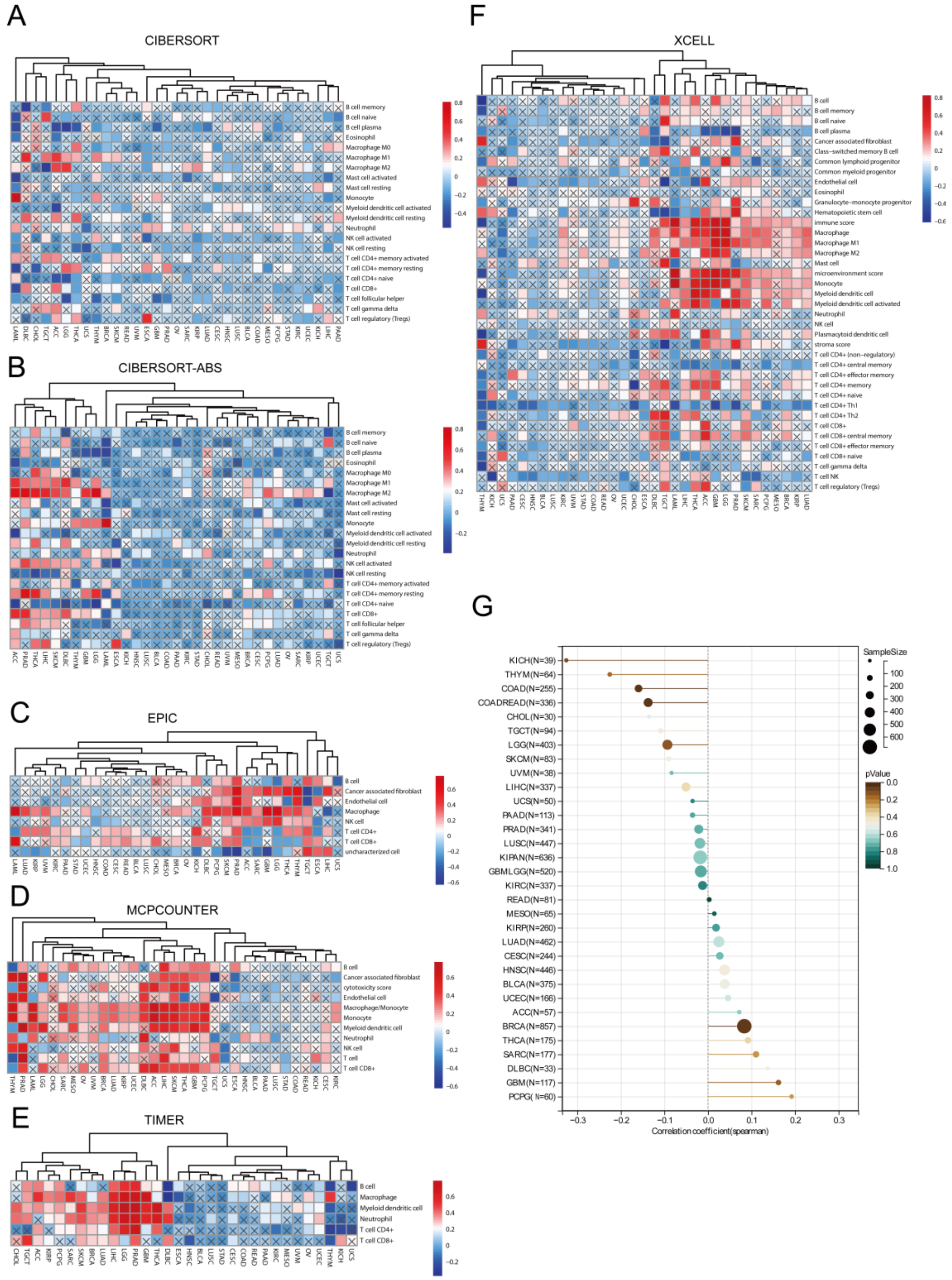

Supplementary Figure 5. The correlation between PLBD1 expression and infiltrated immune cells in pan-cancer. (A-F) The association of PLBD1 expression and immune cells infiltration in pan-cancer with CIBERSORT (A), CIBERSORT-ABS (B), EPIC (C), MCPCOUNTER (D), TIMER (E) and XCELL (F); (G) The association of PLBD1 expression and neoantigens in pan-cancer.

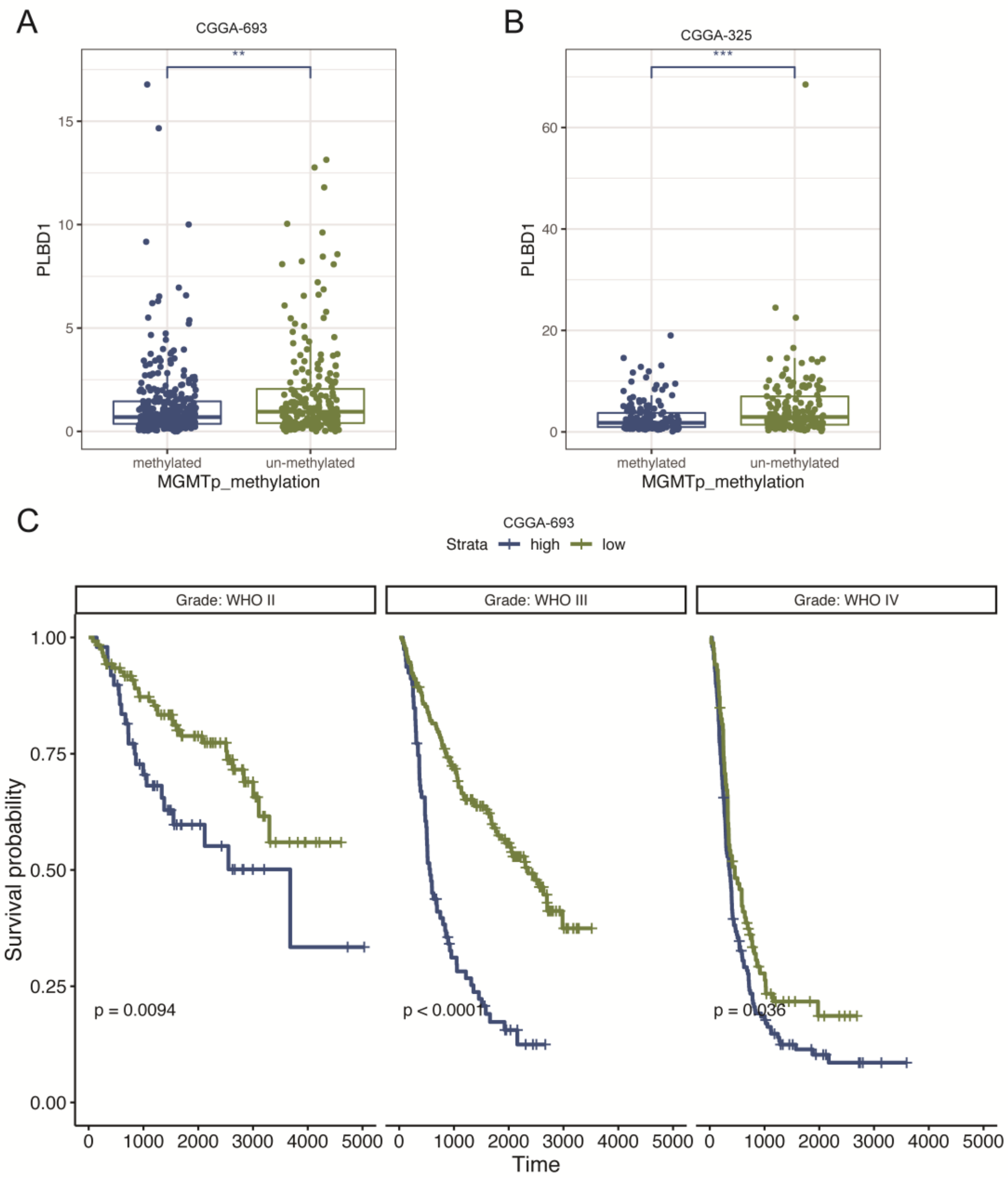

Supplementary Figure 6. The expression and prognostic value of PLBD1 in glioma datasets. (A-B) The mRNA expressions of PLBD1 are shown according to MGMT promoter methylation in CGGA-693 (A) and CGGA-325 (B) datasets; (C) The survival analysis of PLBD1 expression in CGGA-693 according to WHO grades. **p < 0.01; ***p < 0.001.
